# Identification of a novel glycolysis-related gene signature for predicting metastasis and survival in patients with lung adenocarcinoma

**DOI:** 10.1186/s12967-019-02173-2

**Published:** 2019-12-17

**Authors:** Lei Zhang, Zhe Zhang, Zhenglun Yu

**Affiliations:** 1 Department of Breast Surgery, The First Hospital Affiliated China Medical University, No. 155 Nanjing Street, Heping District, Shenyang, 110001 Liaoning China; 2 Department of Thoracic Surgery, The First Hospital Affiliated China Medical University, No. 155 Nanjing Street, Heping District, Shenyang, 110001 Liaoning China

**Keywords:** Lung adenocarcinoma, Glycolysis, Prognostic, mRNAs, Metastasis, Meta-analysis survival

## Abstract

**Background:**

Lung cancer (LC) is one of the most lethal and most prevalent malignant tumors, and its incidence and mortality are increasing annually. Lung adenocarcinoma (LUAD) is the most common pathological type of lung cancer. Several biomarkers have been confirmed by data excavation to be related to metastasis, prognosis and survival. However, the moderate predictive effect of a single gene biomarker is not sufficient. Thus, we aimed to identify new gene signatures to better predict the possibility of LUAD.

**Methods:**

Using an mRNA-mining approach, we performed mRNA expression profiling in large LUAD cohorts (n = 522) from The Cancer Genome Atlas (TCGA) database. Gene Set Enrichment Analysis (GSEA) was performed, and connections between genes and glycolysis were found in the Cox proportional regression model.

**Results:**

We confirmed a set of nine genes (HMMR, B4GALT1, SLC16A3, ANGPTL4, EXT1, GPC1, RBCK1, SOD1, and AGRN) that were significantly associated with metastasis and overall survival (OS) in the test series. Based on this nine-gene signature, the patients in the test series could be divided into high-risk and low-risk groups. Additionally, multivariate Cox regression analysis revealed that the prognostic power of the nine-gene signature is independent of clinical factors.

**Conclusion:**

Our study reveals a connection between the nine-gene signature and glycolysis. This research also provides novel insights into the mechanisms underlying glycolysis and offers a novel biomarker of a poor prognosis and metastasis for LUAD patients.

## Background

Lung cancer (LC) is the leading cause of cancer death worldwide [1, 2]. Cancer metastasis and recurrence remain challenging clinical problems. Although advancements in treatment have been achieved, including improvements in surgery, targeted therapy, chemotherapy and radiotherapy, the 5-year survival rate is only 15% [3, 4]. Lung adenocarcinoma (LUAD) is a pathological subtype of lung cancer with a survival rate of 4–17% [5]. Despite progress in molecular targeted therapy, which has been further developed for years, more targets need to be identified. Some evidence has shown that the discovery and application of molecular biomarkers can provide prognostic value. Thus, for this study, we selected high-risk LUAD patients. Some relevant glycolytic enzymes promote the growth of LUAD cells, and the “Warburg effect” has since been demonstrated in different types of cancer. Therefore, the development of a new glycolysis-related gene signature could predict LUAD.

In recent years, many studies have confirmed that several biomarkers are prognostic factors of lung adenocarcinoma. For example, PKM2 (The M2 isoform of pyruvate kinase) is essential in the metabolism and growth of tumor cells, and increased PKM2 during TGF-β1 (beta-type transforming growth factor mRNA) signaling induces epithelial–mesenchymal transition (EMT) in LUAD cells, which is a biomarker for treatment response [6, 7]. CAV1 (caveolin 1) and DCN (decorin) inhibit LUAD cell proliferation and play an important role in regulating LUAD progression [8]. SPINK1 (serine protease inhibitor Kazal type 1) can promote LUAD cell growth, migration, and invasion [9]. Additionally, miRNAs have been considered new biomarkers of cancer with infinite clinical value because of their remarkable stability in tissues, serum and other body fluids [10]. With the development of high-throughput sequencing, numerous databases have facilitated a detailed understanding of genomic alterations in disease, including the identification of changes in patient genomes by some researchers, and many biomarker changes associated with prognosis and survival have been revealed by mining databases [11]. However, a single gene biomarker cannot produce good predictive effects, and some studies have found that gene signatures are a better alternative for predicting prognosis and survival [12]. Multigene prognostic signatures based on original cancer biopsies can be used in clinical treatment. However, not all pathways have been explored to identify new LUAD biomarkers. Thus, more efforts are needed to find more efficient and sensitive biomarkers for LUAD.

A large amount of data have been generated using special tools. For example, regarding the major public project The Cancer Genome Atlas (TCGA) [13], we used Gene Set Enrichment Analysis (GSEA) to search for some genes and perform further analysis. Generally, some studies have focused on comparing the gene expression of two groups and performed research on some genes that were highly up- or downregulated. Unfortunately, some genes that did not show significant differences but that had important biological significance, information, and connections among gene regulatory networks, gene functions and characteristics were omitted. The benefit of GSEA is that it does not require a distinct differential gene threshold. The algorithm is unique in that genes whose expression is based on the entire trend of effective data and overall level can be identified even without any prior experience. To build a better relationship between the mathematical significance of these data and the biological significance of gene expression, we need to identify additional methods relative to the biomarkers of LUAD.

We aimed to identify gene and pathway signatures with suitable performance to be used in clinical applications, with the goals of providing more insight into tumor cell growth, death and metastasis and opening a new avenue for targeted treatment. From our study, we have drawn hallmark gene sets from 522 LUAD cases with complete mRNA expression datasets from the TCGA database. We have confirmed the key mRNAs related to glycolysis and have built a nine-gene risk signature that can accurately predict patient prognosis. Surprisingly, in several pathways, this glycolysis-related risk signature can successfully predict patients who are in the high-risk group and who have a poor prognosis.

## Methods

### Patient clinical data and mRNA expression dataset

The mRNA expression profiles and clinical data of 522 LUAD patients were extracted from the TCGA database (https://cancergenome.nih.gov/) and divided into two groups: a lymph node metastasis group and a non-lymph node metastasis group. Additionally, the following clinical information was recorded: sex, age, tumor size, number of lymph node metastases, status of distant organ metastasis, neoplasm cancer status, and residual tumor. Finally, 511 patients were classified. The general clinical features are detailed in Table 1.

**Table 1:** Clinical pathological parameters of patients with lung adenocarcinoma in this research

Clinical characteristic	N	%
Age (years)		
> 66	241	46.2
< 66	262	50.2
= 66	19	3.6
Gender		
Male	242	46.4
Female	280	53.6
T classification		
T1−T2	453	86.8
T3–T4	69	13.2
N classification		
N0	325	62.3
N1–3	186	35.6
M classification		
M0	353	67.6
M1–Mx	165	31.6
UICC stage		
I stage	279	53.4
II–IV stage	234	44.8
Neoplasm cancer status		
With tumor	314	60.2
Tumor free	111	21.3
Residual tumor		
R0	348	66.7
R1	44	8.4

### Gene Set Enrichment Analysis

GSEA (http://www.broadinstitute.org/gsea/index.jsp) was performed to explore whether the identified gene sets showed significant differences between the groups [14]. The expression levels of 24,991 mRNAs were analyzed between the lymph node metastasis and non-lymph node metastasis groups. Normalized P values (P < 0.05) were used to determine which functions could be used for further investigation.

### Statistical analysis

The expression profiles of 24,991 mRNAs are shown as raw data, and each mRNA was log2 normalized for further analysis. Cox regression was used to analyze and identify genes with obvious relationships to metastasis and OS with P values < 0.05. Next, we used multivariate Cox proportional hazards regression to analyze and further confirm the prognostic genes from the previous steps. The filtered mRNAs were classified into risk (hazard ratio (HR) > 1) and protective (0 < HR < 1) types. Thereafter, a prognostic risk score formula was established based on a linear combination of expression levels weighted with the regression coefficients derived from the multivariate Cox regression analysis. Risk score = expression of gene 1 × β1 + expression of gene 2 × β2 +⋯+ expression of gene n × βn. We separated 522 patients into high-risk and low-risk subgroups by the median value using the median risk score as the cutoff. Next, we used Kaplan–Meier curves and log-rank methods to validate the prognostic importance of the risk score. Subsequently, we examined the differential expression of optimal genes between the lymph node metastasis and non-lymph node metastasis groups. We classified them into high-risk and low-risk groups by the median risk score and used the KM method (multiplication of the positive limit) to predict the accuracy of the survival status and survival time. The survival function was constructed by the KM method, and the ROC curve was drawn. Additionally, we conducted univariate Cox regression and multivariable Cox regression analyses to check whether the risk score was a prognostic factor within the available data. Select gene alterations are shown online for specific cancer types (http://www.cbioportal.org/). All statistical analyses were performed using SPSS 16.0 and Graph Pad Prism 7 software.

## Results

### Initial screening of genes using GSEA

**Fig. 1:**
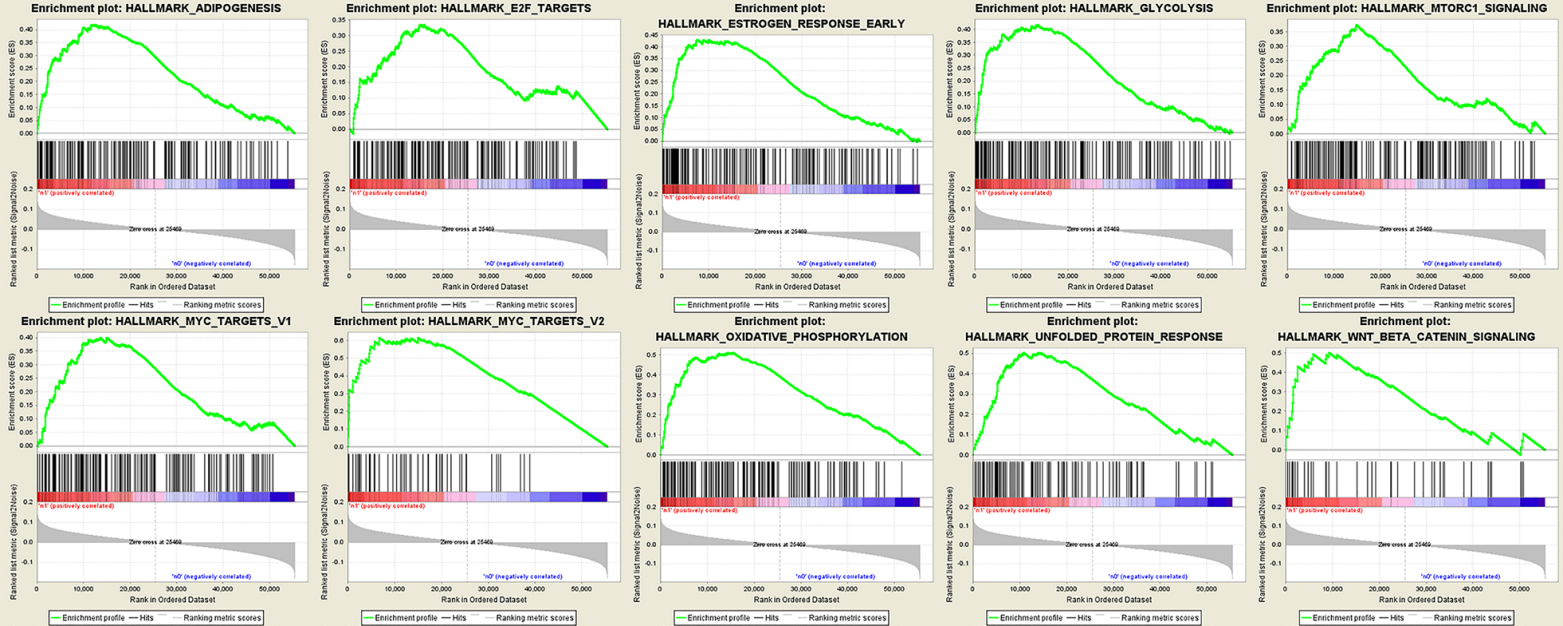
Enrichment plots of nine gene sets which were importantly differentiated between in lymphonode metastasis and non-lymph node metastasis tissues

**Fig. 2:**
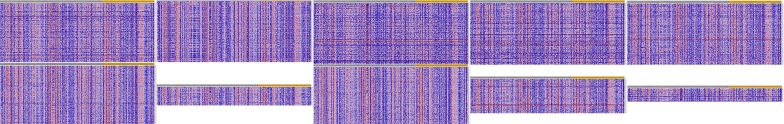
Selected genes’ sets in nine genes

We obtained clinical features from 522 patients with LUAD, along with an expression data set for 24,991 mRNAs, from the TCGA database. The expression signatures of the hallmark gene sets, each containing 50 specific gene sets, were derived by concentrating multiple gene sets from the Molecular Signatures Database (MSigDB) to represent well-defined biological statuses or courses. GSEA was performed using the above detailed data to detect whether the identified gene sets showed statistically notable differences between the lymph node metastasis and non-lymph node metastasis groups. Fifty gene sets were upregulated in lung adenocarcinoma, and 10 gene sets, namely, oxidative phosphorylation, the MYC target V2, unfolded protein response, estrogen early response, adiposeness, glycolysis, the MYC target V1, mtorc1 signaling, wnt beta catenin signaling, and E2F targets, were greatly enriched, with normalized *P* values < 5% among the 50 gene sets (Figs. 1 and 2). Additionally, we selected the top-ranking function, namely, glycolysis (*P *= 0), which contained 198 genes and was the largest in size.

### Identification of glycolysis-related mRNAs associated with metastasis and patient survival

**Fig. 3:**
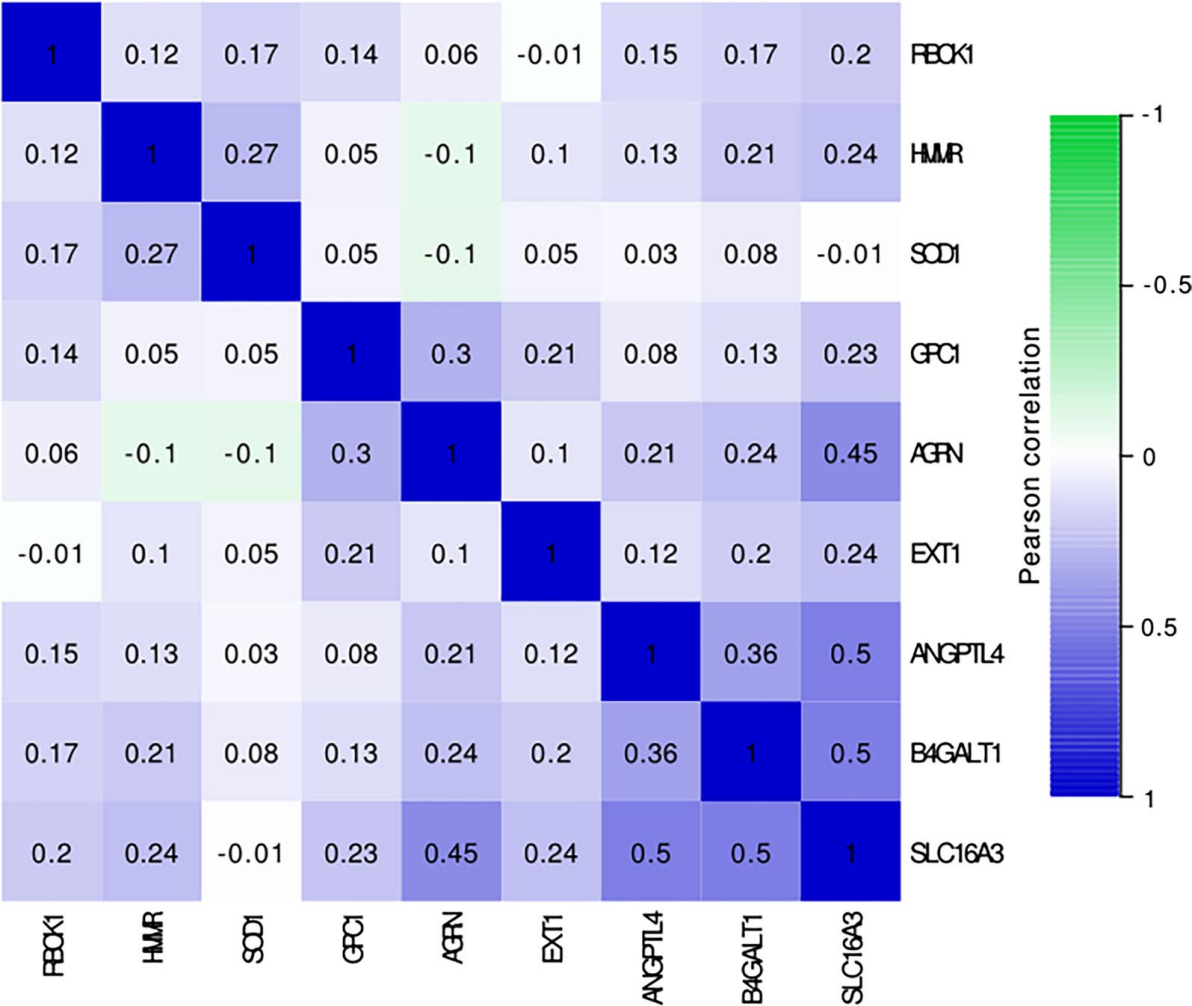
Correlations between the expression levels of nine genes in lung adenocarcinoma were evaluated with the Pearson correlation coefficient

First, we employed univariate Cox regression analysis of the 86 genes for preliminary screening and obtained 30 genes with p values < 0.1. Additionally, multivariate Cox regression analysis was used to further examine the relationship between the expression profiles of 30 mRNAs and the patient survival rate. Subsequently, 9 mRNAs (HMMR, B4GALT1, SLC16A3, ANGPTL4, EXT1, GPC1, RBCK1, SOD1, and AGRN) were verified as independent indicators of poor prognosis and metastasis. The filtered mRNAs were classified into a risk type (HMMR, B4GALT1, ANGPTL4, EXT1, GPC1, RBCK1, SOD1, and AGRN), whose HR was > 1 with metastasis, and a protective type (SLC16A3), whose HR was < 1 with nonmetastasis (Table 2). We calculated the Pearson correlation coefficient among the 9 mRNAs on the basis of Table 2, and we found correlations between B4GALT1 and SLC16A3, between B4GALT1 and ANGPTL4, between SLC16A3 and ANGPTL4, between SLC16A3 and AGRN, and between GPC1A and GRN, with an R^2^ value greater than 0.3 (Fig. 3).

**Table 2:** The information of nine prognostic mRNAs importantly associated with metastasis and overall survival in patients with lung adenocarcinoma

mRNA	Ensemble ID	Location	B (cox)	HR (95% CIs)	P
HMMR	ENSG00000072571	Chr5: 163,460,203–163,491,945	0.2551	1.3060	0.00025
B4GALT1	ENSG00000086062	Chr9: 33,104,082–33,167,356	0.2160	1.2411	0.07121
SLC16A3	ENSG00000141526	Chr17: 82,228,397–82,261,129	− 0.1570	0.8547	0.13969
ANGPTL4	ENSG00000167772	Chr19: 8,363,289–8,374,373	0.1238	1.1318	0.01137
EXT1	ENSG00000182197	Chr8: 117,794,490–118,111,853	0.2381	1.2688	0.04074
GPC1	ENSG00000063660	Chr2: 240,435,671–240,468,078	0.1027	1.1082	0.15791
RBCK1	ENSG00000125826	Chr20: 407,498–430,966	0.1820	1.1996	0.12534
SOD1	ENSG00000142168	Chr21: 31,659,622–31,668,931	0.1874	1.2061	0.11555
AGRN	ENSG00000188157	Chr1: 1,020,123–1,056,118	0.2226	1.2494	0.02277

**Fig. 4:**
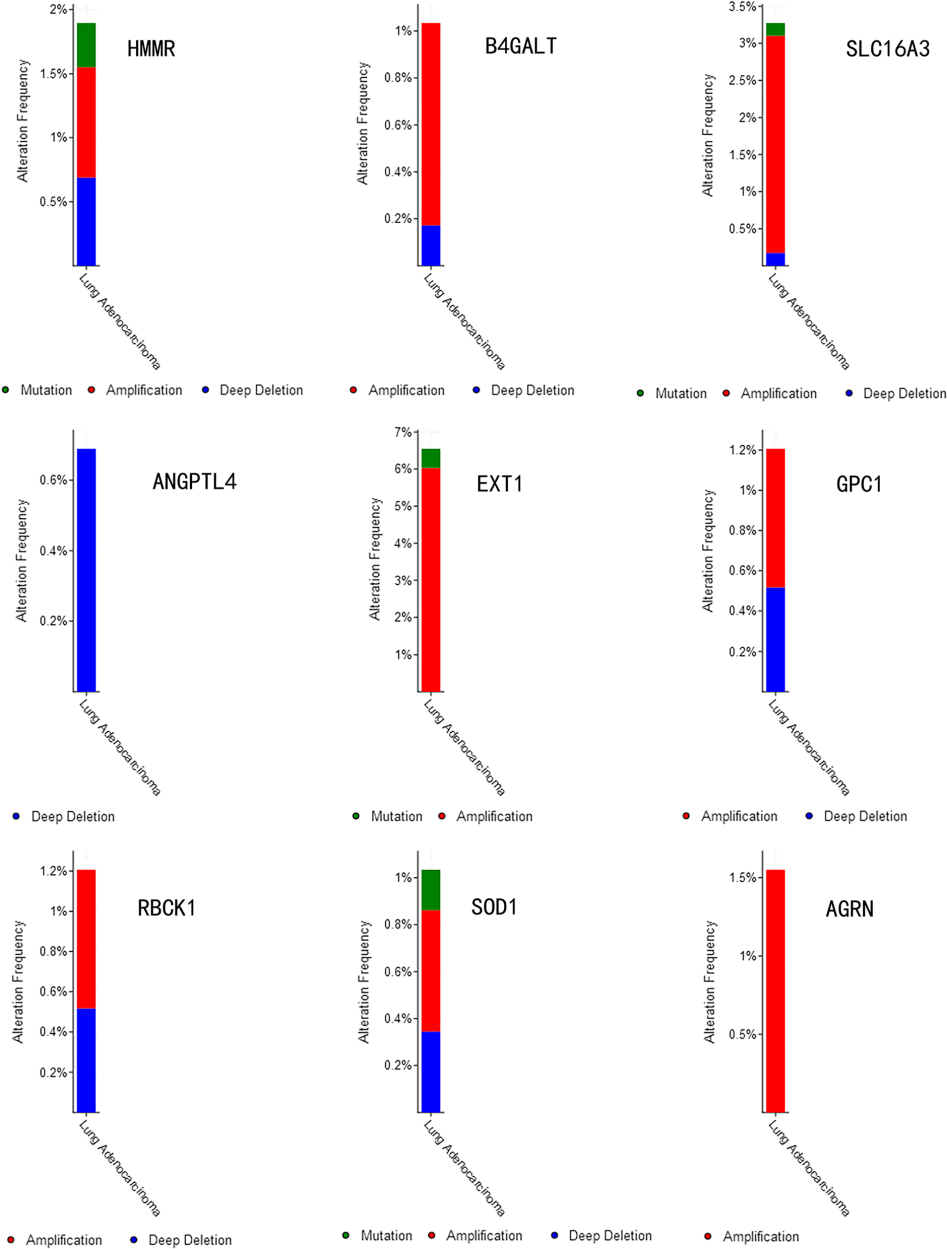
Selected genes’ specific alteration frequency with the study of clinical samples

Specific alterations in the selected genes were also clear in specific cancer types. Among the 522 patients with lung adenocarcinoma, 0.18% had mutations, 0.4% had amplifications, and 0.35% had deep deletions in HMMR; 0.68% had mutations and 0.52% had deep deletions in B4GALT1; 0.68% had mutations and 0.52% had deep deletions in SLC16A3; 0.5% had mutations and 6% had amplifications in ANGPTL4; 6.4% had deep deletions in EXT1; 0.2% had mutations, 2.9% had amplifications, and 0.2% had deep deletions in GPC1; 0.88% had amplifications and 0.16% had deep deletions in RBCK1; 0.25% had mutations, 0.85% had amplifications, and 0.7% had deep deletions in SOD1; and 1.51% had amplifications in AGRN (Fig. 4).

### Construction of the nine-mRNA signature to predict patient outcomes

**Fig. 5:**
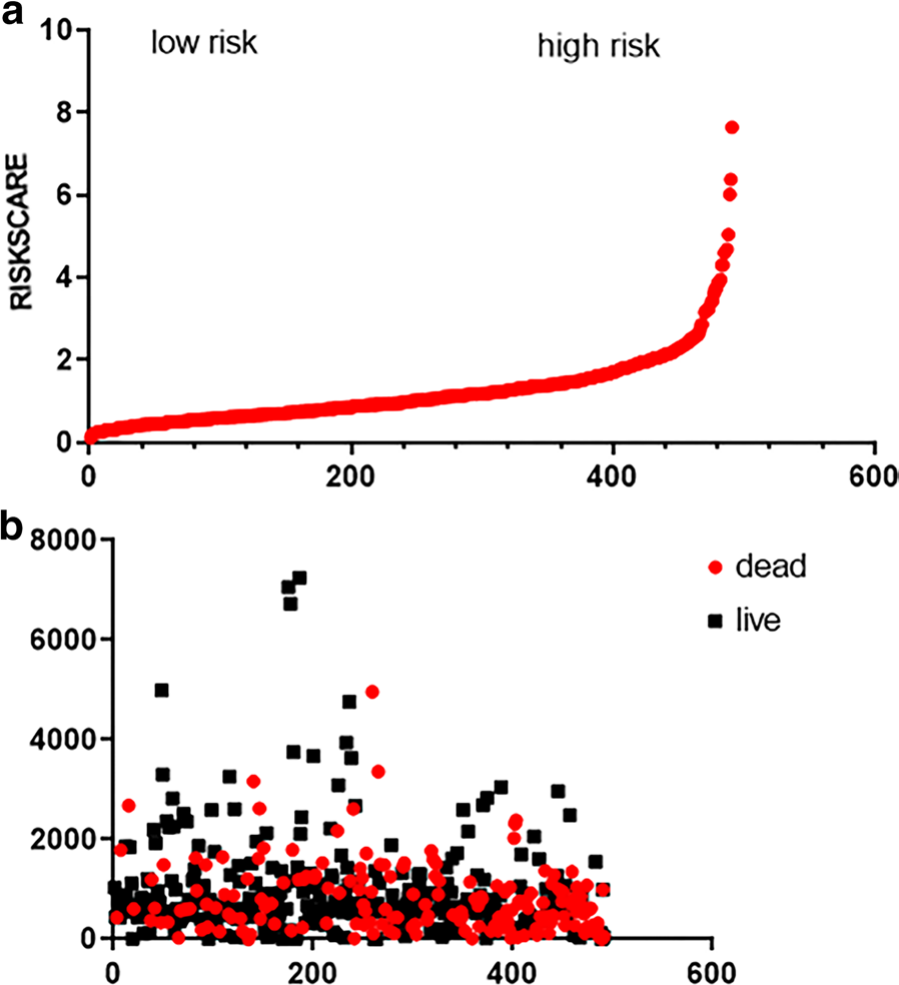
The nine-mRNA signature related to risk score predicts overall survival in the patients with lung adenocarnicoma. **a** mRNA risk score distribution. **b** Survival days of patients

**Fig. 6:**
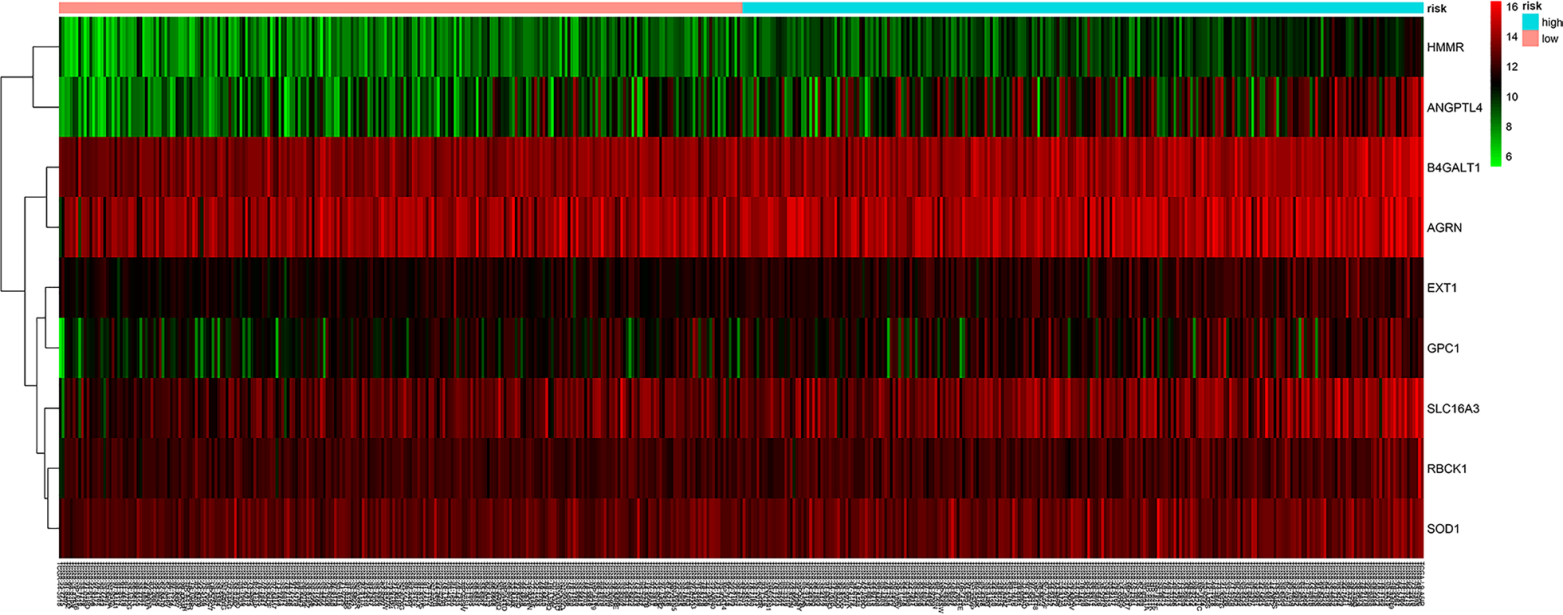
Heatmap of nine genes’ expression profile. The figure shows that the expression of each gene is significantly different between high and low-risk groups

**Fig. 7:**
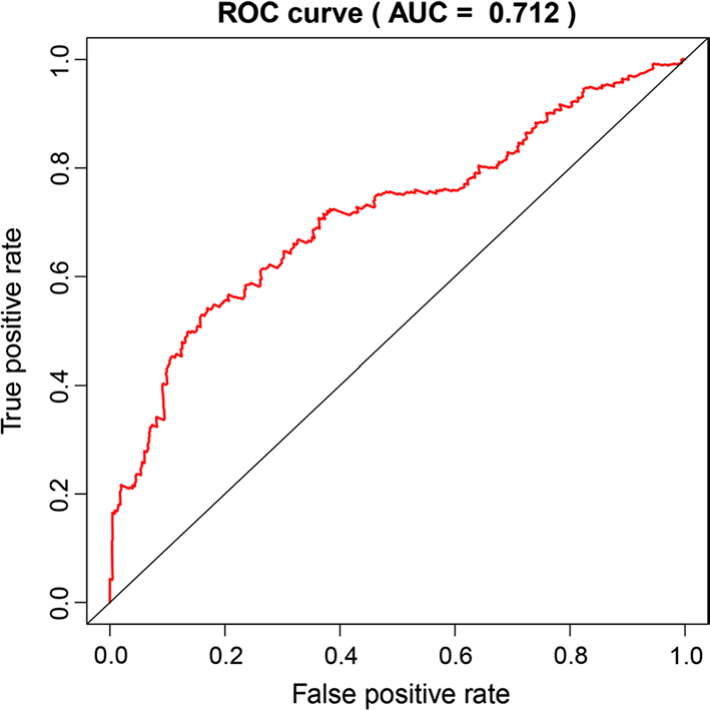
Receiver operating characteristic (ROC) analysis of the sensitivity and specificity of the risk score model

A prognostic risk score formula was established based on a linear combination of the expression levels weighted with the regression coefficients derived from multivariate Cox regression analysis: Risk score = 0.2551 × expression of HMMR + 0.2160 × expression of B4GALT1-0.1570 × expression of SLC16A3 + 0.1238 × expression of ANGPTL4 + 0.2381 × expression of EXT1 + 0.1027 × expression of GPC1 + 0.1820 × expression of RBCK1 + 0.1874 × expression of SOD1 + 0.2226 × expression of AGRN. Each patient with LUAD lymph node metastasis had only one risk score. We calculated the scores, ranked them and then classified the patients into high- and low-risk groups by the median value (Fig. 5a). The survival time (in days) of each patient is shown in Fig. 5b, and the patients with high-risk scores showed higher mortality rates than those with low-risk scores. Additionally, a heatmap (Fig. 6) was revealed to display the expression profiles of the nine mRNAs, and the 9-mRNA expression-based survival risk score was used to assign patients into a low-risk or high-risk group using the median risk score as the cut-off. The ROC curve analysis score was 0.712 (Fig. 7), indicating the good sensitivity and specificity of the 9-mRNA signature in predicting metastasis and survival in LUAD patients. With the increasing risk score of patients with lymph node metastasis of lung adenocarcinoma, the expression of high-risk mRNAs (HMMR, B4GALT1, ANGPTL4, EXT1, GPC1, RBCK1, SOD1, AGRN) was obviously upregulated. In contrast, the expression of the protective type of mRNAs (SLC16A3) was downregulated.

### Generation of the risk score from the nine-mRNA signature as an indicator of metastasis and prognosis

**Fig. 8:**
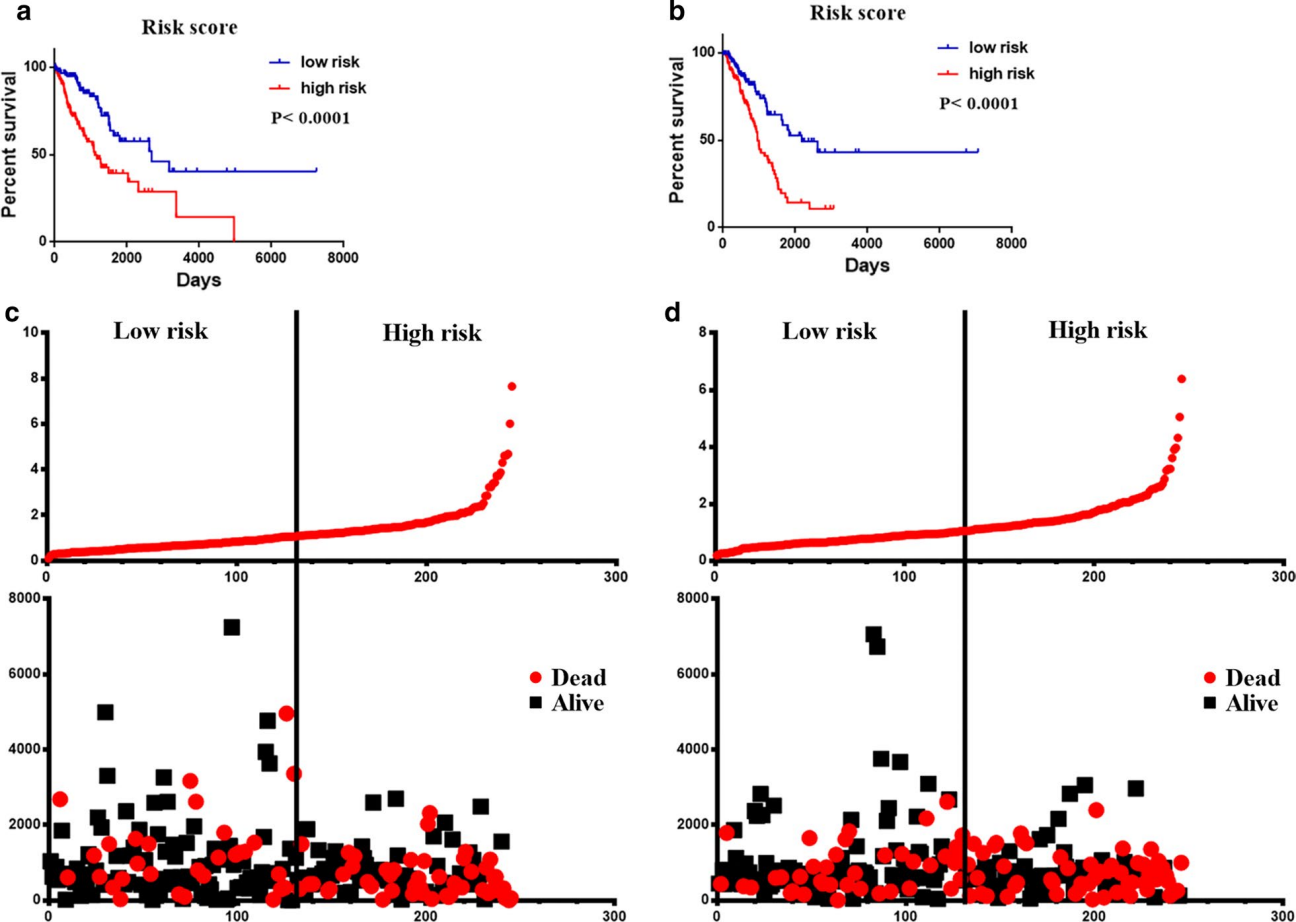
Group 1: **a** The risk score of nine-mRNA signature **c** mRNA risk score distribution and survival days of patients. Group 2: **b** The risk score of nine-mRNA signature (**d**)

The prognostic values of the risk scores were compared with the clinicopathological information by univariate and multivariate analyses. Samples with completed clinical data were used for analysis. The 522 patients with lung adenocarcinoma had a median age 66 years and comprised 242 male patients and 280 female patients. Among 425 patients, 314 (60.2%) had a positive tumor finding during the follow-up visit. Among 392 patients, 44 (8%) had residual tumors. Among 511 patients, 186 (35.6%) had lymph node metastasis, and 165 (31.6%) had distant metastases. Additionally, we found that the risk score, T classification, N classification, UICC stage, neoplasm cancer status, and residual tumor were independent prognostic indicators because they showed important differences in the univariate analysis, with P values < 0.05 (Table 3). In the subsequent multivariate analysis (Table 3), we found that the risk score and neoplasm cancer status showed statistical significance in univariate and multivariate analyses (P < 0.05). Regardless of the analysis type (univariate or multivariate), the risk score had prominent prognostic values. The result of univariate Cox regression indicated that the risk score was significantly associated with metastasis and prognosis [high-risk group vs low-risk group, HR = 2.017, 95% CI (confidence interval) 1.973–3.698, P < 0.001]. Additionally, in multivariable Cox regression, the risk score also has a significant relationship with metastasis and prognosis (HR = 2.381, 95% CI 1.563–3.628, P < 0.001). And with the increase of risk scores, the expression level of SLC16A3 decreased, whereas the expression levels of HMMR, B4GALT1, ANGPTL4, EXT1, GPC1, RBCK1, SOD1, and AGRN were upregulated. Meanwhile, the number of patient deaths increased. Additionally, the most obvious clinical parameter to predict patient survival was “neoplasm cancer status”, and patients with tumors had a 4.983 times higher chance of death than those who were tumor-free. In order to testify the result of our research, we divided 522 LUAD patients into two groups (group 1 and group 2) randomly, and proved the result of the nine-gene signature combination in each group. The result was the value of risk score is meaningfully in each group (Fig. 8). And we also testify the result of our research in the trial, then we selected the carcinoma tissue of 9 lung adenocarcinoma patients with lymph node metastasis and 9 lung adenocarcinoma patients with not lymph node metastasis, then compared the expression of 9-mRNA gene in these tissues. And the 9-mRNA gene was verified in 18 cases by the qRT-PCR (Additional file 1: Fig. S1).We can draw that the result of our research is reasonable between the carcinoma tissue with lymph node metastasis and with not lymph node metastasis.

**Table 3:** Univariable and multivariable analyses for each clinical feature

Clinical feature	Univariate analysis		P	Multivariate analysis		P
	HR	95% CI		HR	95% CI	
Risk score	2.701	1.973–3.698	0.000	2.381	1.563–3.628	0.000
Age	1.076	0.798–1.450	0.632	–	–	–
Gender	1.069	0.797–1.434	0.656	–	–	–
Smoking status	1.207	0.883–1.649	0.238	–	–	–
T classification	1.610	1.142–2.269	0.007	0.985	0.616–1.575	0.985
N classification	2.597	1.932–3.490	0.000	1.209	0.669–2.187	0.530
UICC stage	2.889	2.118–3.940	0.000	1.646	0.850–3.190	0.140
Neoplasm cancer status	4.983	3.528–7.039	0.000	3.557	2.381–5.315	0.000
Residual tumor	2.159	1.318–3.536	0.002	1.203	0.676–2.144	0.530

### Validation of nine mRNA markers for survival prediction by Kaplan–Meier curve analysis

**Fig. 9:**
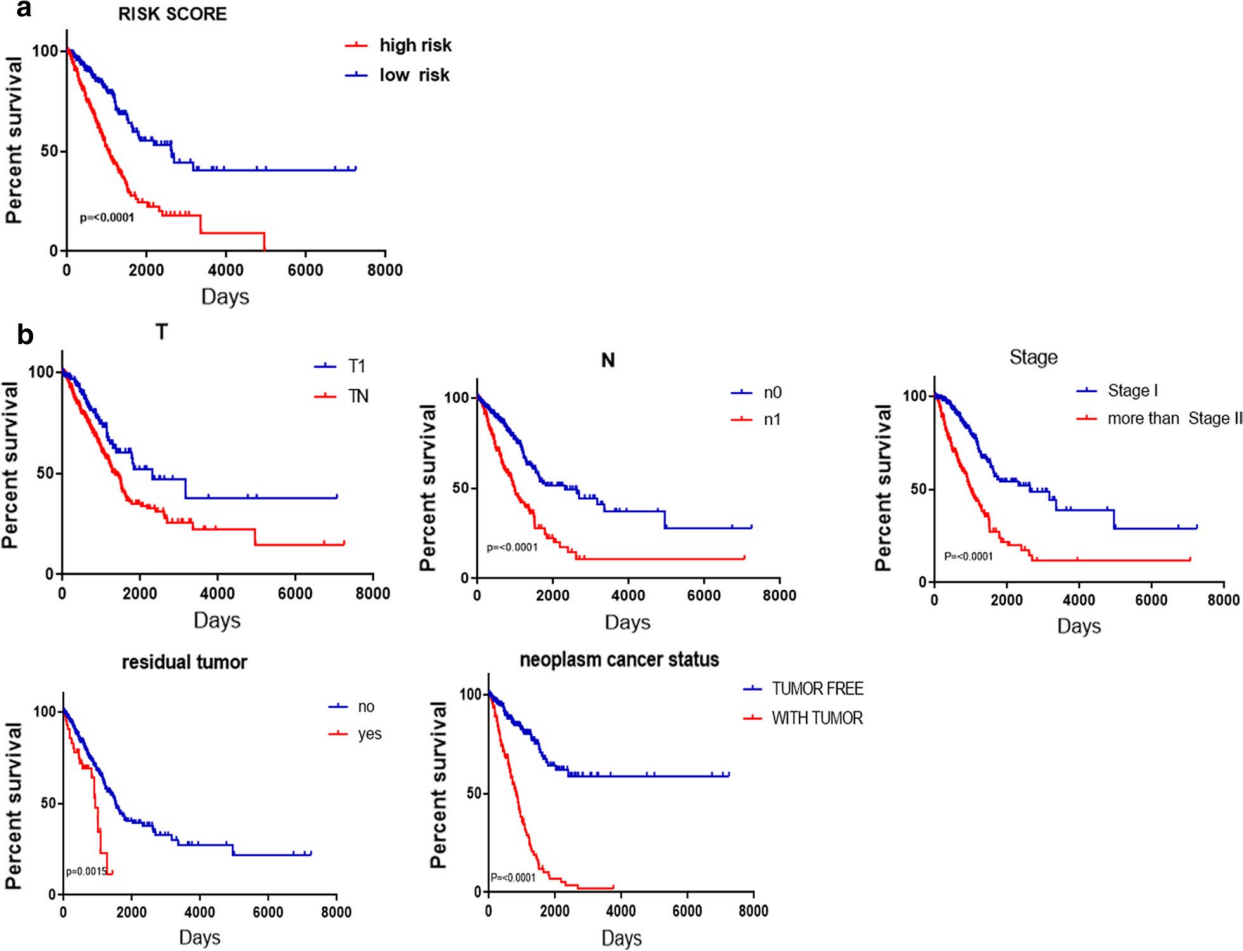
Kaplan–Meier survival analysis for the patients with lung adenocarcinoma in TCGA dataset. **a** The Kaplan–Meier curve for patients divided into high-risk and low-risk. **b** Different clinical features predict patients' survival

**Fig. 10:**
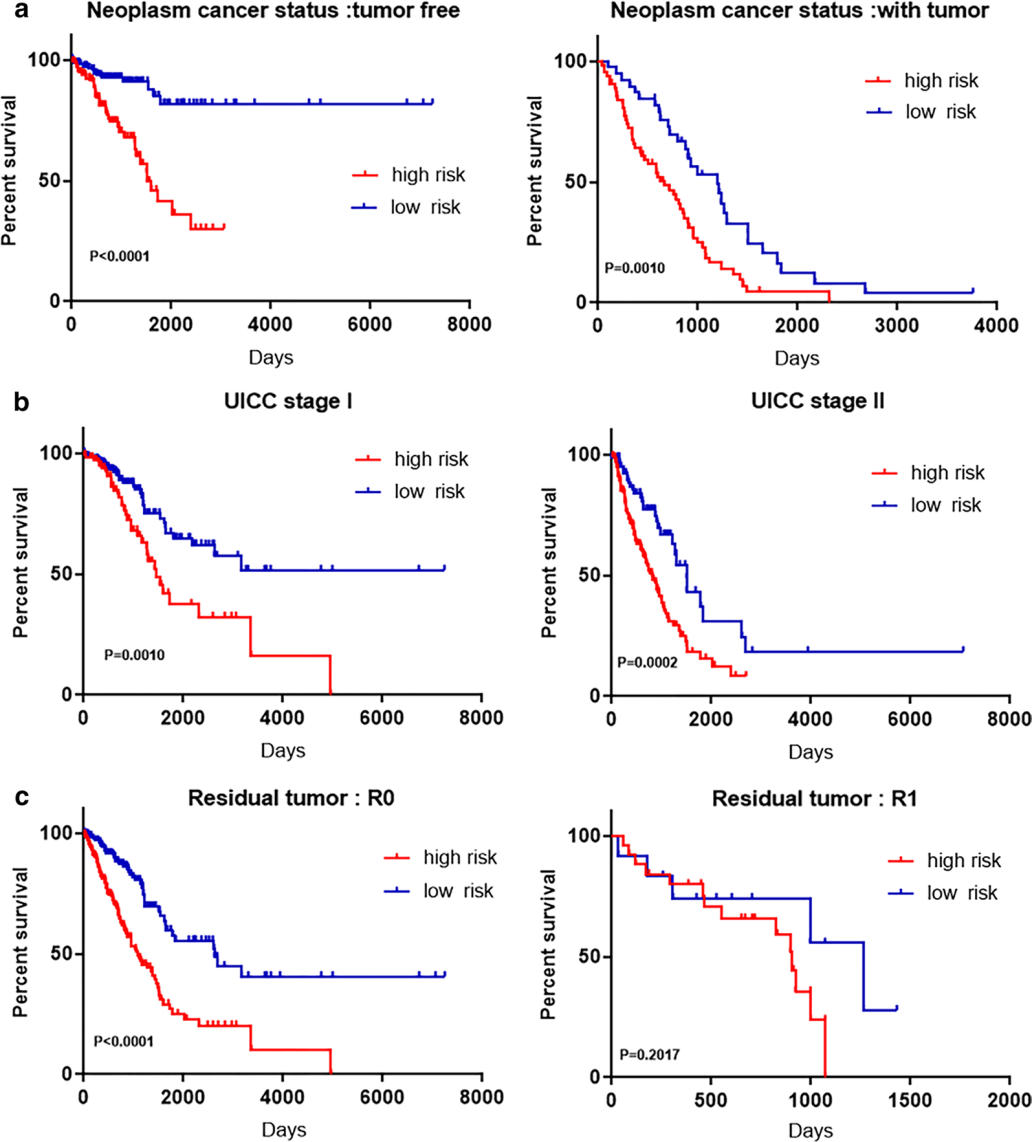
Kaplan–Meier curves for prognostic value of risk-score signature for the patients divided by each clinical features. **a** Neoplasm cancer status **b** UICC stage **c** Residual tumor

The results of Kaplan–Meier survival curves and the log-rank method showed a poor prognosis in patients with high-risk scores (P < 0.0010) (Fig. 9a). A univariate Cox regression analysis of OS showed that several clinicopathological data were effective at predicting the survival rate of lung adenocarcinoma patients, including their T classification, N classification, UICC stage, neoplasm cancer status, and residual tumor status. The K–M method was then adopted to confirm the above results. According to the survival curves, patients with lymph node metastasis, a residual tumor, a tumor diameter greater than 3 cm, a UICC stage greater than stage I, or a positive tumor finding during the follow-up visit were correlated with a poor prognosis (Fig. 9b). These results provided further confirmation of the accuracy of our analysis. Hence, further stratified analysis was performed for data mining. As shown from the K–M curve, regardless of neoplasm status, tumor status (Fig. 10a), or UICC stage (for example, stage I or stage II; Fig. 10b), the nine-mRNA signature was a stable prognostic marker for lung adenocarcinoma patients who were in the high-risk group and had a poor prognosis. However, when considering the different subtypes, based on the residual tumor, the risk score of the nine-mRNA signature was still an independent prognostic indicator for the subgroups with no residual tumor findings (Fig. 10c).

## Discussion

In recent years, some researchers have verified that the clinicopathological features of age, sex, smoking history, tumor size, pathological stage, lymph node metastasis and distant organ metastasis have significant implications for accurately predicting patient prognosis. Therefore, increasingly more mRNAs have the potential to be molecular biomarkers for evaluating and predicting the prognosis of LUAD [15],indicating their considerable clinical significance in research [16]. For example, Zhang et al. reported that the expression of SBP1 is significantly downregulated in intrahepatic cholangiocarcinoma (ICC) and can be considered a prognostic predictor or potential target for ICC treatment [17]. Li et al. confirmed that micro-RNA 145 shows higher expression in hepatocellular carcinoma (HCC) and is an independent prognostic factor for OS using the Cox proportional hazards model [18]. However, patient survival could not be predicted by these genes because various factors can modulate a single gene, resulting in an inaccurate predictive effect. Thus, a gene signature comprising various genes has been built from the statistical model to predict cancer outcomes. The results highlight that the predictive effects of each gene involved can offer a more accurate prediction than a single biomarker [19].

With the development of gene signatures, the prognosis of some types of cancer has been predicted by statistical models. DCTN1, DCTN2, and DCTN4 could serve as biomarkers to predict the prognosis and diagnosis of colon adenocarcinoma (COAD) [20]. A novel prognostic biomarker of ovarian cancer was searched and involved a five-DNA methylation marker signature through Cox regression and ROC analyses [21]. We also performed GSEA analysis using the mRNA expression data of the 522 LUAD patients and found that 9 exhibit significant differences, with a P value < 0.05, and the minimum P value could be used for further analysis. We explored specific functions to identify genes by GSEA that could predict the survival of LUAD patients. Furthermore, we identified a combination of 9 genes with prognostic value for LUAD patients instead of a single gene by univariate Cox regression and multivariate Cox regression analyses. Subsequently, through comparison with some known prognostic biomarkers, we found that our z identified risk signature may strongly support clinical results. We analyzed glycolysis-related genes using the LUAD dataset in TCGA and then compared the lymph node data with the non-lymph node data of LUAD patients. Kaplan–Meier analysis showed that a high-risk score was associated with metastasis and a poor prognosis. Among our 9 genes, HMMR has been found to be involved in many human solid carcinomas, including breast, non-small-cell lung, prostate, bladder, ovarian, and colorectal cancers, contributing to disease progression, aggressive phenotypes, and a poor prognosis in these patients. Zhang et al. showed that high expression of *HMMR* is significantly correlated with tumor relapse, predicts a poorer prognosis and induces EMT in gastric cancer patients [22]. B4GALT1 is a β-1,4-galactosyltransferase that catalyzes the transfer of galactose from the sugar nucleotide donor uridine diphosphate galactose to glycoside residues with a terminal *N*-acetylglucosamine (GlcNAc) moiety. Poeta et al. demonstrated that the glycogene *B4GALT1* is a valuable candidate biomarker of the invasive phenotype of colorectal *cancer* [23]. ANGPTL4 was found to promote gastric cancer proliferation and metastasis [24]. Exostoxin 1 (EXT1) is an endoplasmic reticulum (ER)-residing type II transmembrane glycoprotein involved in the biosynthesis of cell surface heparin sulfate (HS). EXT1 promotes epithelial–mesenchymal transition (EMT) and migratory behavior in breast cancer cells [25]. Whipple et al. reported that GPC1 plays an important role in tumor development and metastasis in pancreatic ductal adenocarcinoma [26]. SOD1 is a soluble Cu/Zn enzyme that is mainly located in the cytosol, although a small percentage of SOD1 proteins (~ 3%) is found in the intermembrane space of the mitochondria; SOD1 was found to decrease pso-mediated ROS in prostate cancer, inducing tumor cell growth and metastasis [27]. Agrin (AGRN) is a multifunctional heparan sulfate proteoglycan of the extracellular matrix that is localized in the basement membrane of the vessels and ducts. Wu et al. demonstrated that high levels of AGRN are related to the metastasis and poor prognosis of papillary thyroid carcinoma (PTC) [28]. However, we found no relationship with metastasis for SLC16A3 and RBCK1. Conventional prognostic systems generally make inaccurate predictions for risk stratification and estimations of clinical outcomes because of the heterogeneity between patients. To the best of our knowledge, compared with a single common biomarker, the 9-mRNA signature can better predict the metastasis and prognosis of lung adenocarcinoma.

Increased glycolysis, which is also called the “Warburg effect” [29, 30], has been found in several types of cancer and facilitates metastatic dissemination [31]. MiRNAs play an important role in the process of regulating glycolysis in cancer cells; for example, miR-143 can increase glucose metabolism and promote cell proliferation by targeting HK II directly in lung tumors [32]. Sinthupibulyakit et al. revealed that 2-deoxy-d-glucose (2DG) has a cytotoxic effect on NSCLC that is p53 dependent [33]. Farah et al. showed that inhibitors of glycolysis could regulate the cell survival of LUAD and function as an indicator for lung cancer treatment [34]. Kayser et al. showed that TKTL1, a regulator of glycolysis, is expressed in NSCLC and serves as a new biomarker of pathology [35]. Altenberg and Greulich demonstrated that various enzymes of glycolysis are upregulated in lung carcinoma [36]. Hexokinase (HK) [37] is an important enzyme in glycolysis that accelerates the rate of glycolysis and regulates tumor survival. In 1999, HK was identified by Katabi, who revealed that HIF-1α can facilitate the activity of the glycolysis pathway by regulating HK I in a LUAD cell line [38]. Phosphofructokinase (PFK) is also a key enzyme in glycolysis [39]. High levels of PFK mRNA in human lung cancer tissues and A549 cells were found compared with normal tissue. Additionally, PFK isozymes are highly induced in lung adenocarcinoma cells under hypoxic conditions [40]. Pyruvate kinase (PK) [39] is the last enzyme in glycolysis. Parnell et al. used small-molecule PKM2 activators to affect the growth of LUAD cells in vitro and in vivo by raising the affinity of PKM2 and PEP [41]. Overall, these important enzymes of glycolysis play significant roles in the proliferation and growth of LUAD cells; therefore, glycolysis may be involved in the development and progression of LUAD. A single gene related to glycolysis has been reported to predict the prognosis of LUAD, but no glycolysis-related gene signatures have been established. In this work, we first reported a gene signature (HMMR, B4GALT1, SLC16A3, ANGPTL4, EXT1, GPC1, RBCK1, SOD1, and AGRN) related to glycolysis and then demonstrated the prognostic value of this gene signature for LUAD.

In conclusion, this work is the first to report a nine-gene risk signature related to glycolysis that can help predict survival and metastasis in LUAD patients. A higher risk score indicates a poorer prognosis. This finding will help future researchers in their efforts to identify new treatments for LUAD and to provide more gene targets to cure LUAD in patients.

## Conclusion

We used a nine-gene signature (HMMR, B4GALT1, SLC16A3, ANGPTL4, EXT1, GPC1, RBCK1, SOD1, AGRN) to predict and evaluate LUAD via tissue or blood samples and examined whether mutations in these genes can promote the development of LUAD. Furthermore, we identified treatments related to glycolysis to successfully target these genes; this signature could also be used to develop new targeted treatments to cure LUAD patients. Finally, we confirmed the relationship between the nine-gene risk signature related to glycolysis and LUAD.

## Supplementary information

**Additional file 1: Fig. S1.** The different expression of 9 mRNAs (HMMR, B4GALT1, SLC16A3, ANGPTL4, EXT1,GPC1, RBCK1, SOD1, and AGRN) in lung adenocarcinoma tissue with lymph node metastasis and not lymph node metastasis. *P < 0.05.

## Data Availability

All data generated or analyzed during this study were included in this published article and its additional files.

## Abbreviations

LC: lung cancer
LUAD: lung adenocarcinoma
TCGA: The Cancer Genome Atlas
GSEA: Gene Set Enrichment Analysis
OS: overall survival
HR: hazard ratio
MSigDB: Molecular Signatures Database
UICC: Union for International Cancer Control
EMT: epithelial–mesenchymal transition
CI: confidence interval
ICC: intrahepatic cholangiocarcinoma
HCC: hepatocellular carcinoma
COAD: colon adenocarcinoma
2DG: 2-deoxy-d-glucose
PTC: papillary thyroid carcinoma
HK: hexokinase
PFK: phosphofructokinase
PK: pyruvate kinase
