# Supplementary figures and images for: Identification of a novel glycolysis-related gene signature for predicting metastasis and survival in patients with lung adenocarcinoma
Source: J Transl Med. 2019 Dec 17;17:423. doi: 10.1186/s12967-019-02173-2 (PMC6916245; doi:10.1186/s12967-019-02173-2)

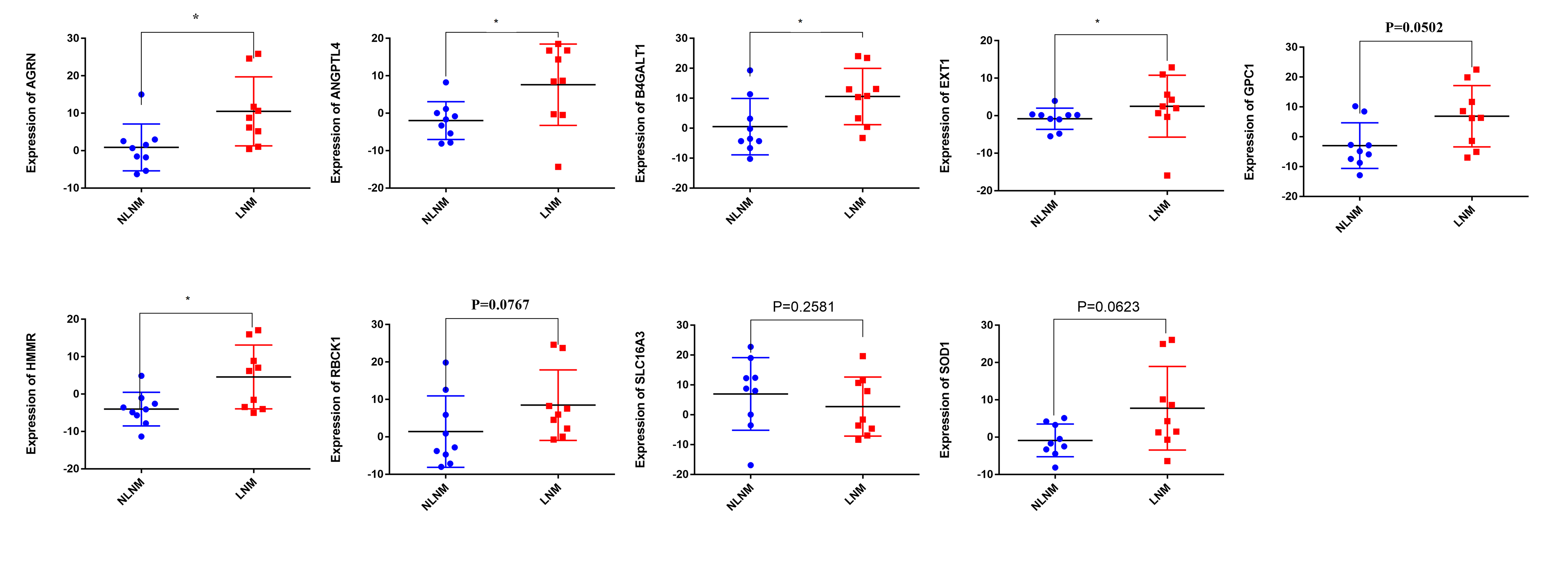

Supplement: Supplementary file 1 — Additional file 1: Fig. S1. The different expression of 9 mRNAs (HMMR, B4GALT1, SLC16A3, ANGPTL4, EXT1,GPC1, RBCK1, SOD1, and AGRN) in lung adenocarcinoma tissue with lymph node metastasis and not lymph node metastasis. *P < 0.05. [file 12967_2019_2173_MOESM1_ESM.tif]
